# Metabolomics-driven strain improvement: A mini review

**DOI:** 10.3389/fmolb.2022.1057709

**Published:** 2022-11-09

**Authors:** Marvin Nathanael Iman, Elisa Herawati, Eiichiro Fukusaki, Sastia Prama Putri

**Affiliations:** ^1^ Department of Biotechnology, Graduate School of Engineering, Osaka University, Osaka, Japan; ^2^ Department of Biology, Faculty of Mathematics and Natural Sciences, Universitas Sebelas Maret, Surakarta, Indonesia

**Keywords:** metabolomics, metabolic engineering, strain improvement, enzyme engineering, bioproductivity

## Abstract

In recent years, mass spectrometry-based metabolomics has been established as a powerful and versatile technique for studying cellular metabolism by comprehensive analysis of metabolites in the cell. Although there are many scientific reports on the use of metabolomics for the elucidation of mechanism and physiological changes occurring in the cell, there are surprisingly very few reports on its use for the identification of rate-limiting steps in a synthetic biological system that can lead to the actual improvement of the host organism. In this mini review, we discuss different strategies for improving strain performance using metabolomics data and compare the application of metabolomics-driven strain improvement techniques in different host microorganisms. Finally, we highlight several success stories on the use of metabolomics-driven strain improvement strategies, which led to significant bioproductivity improvements.

## Introduction

As the use of biological systems to manufacture industrially relevant products is becoming more mainstream, the search for reliable strategies to improve bioproduction capabilities of chassis organisms becomes crucial. Traditionally, attempts to improve strain bioproductivity have often been confined to the trial-and-error-based modifications of single genes without a systemic understanding of the metabolism of the chassis (Opgenorth et al., 2019). Recent advancements in omics technologies, including mass spectrometry-based metabolomics, have opened the door to a more systemic approach of metabolic engineering (Amer and Baidoo, 2021).

**FIGURE 1:**
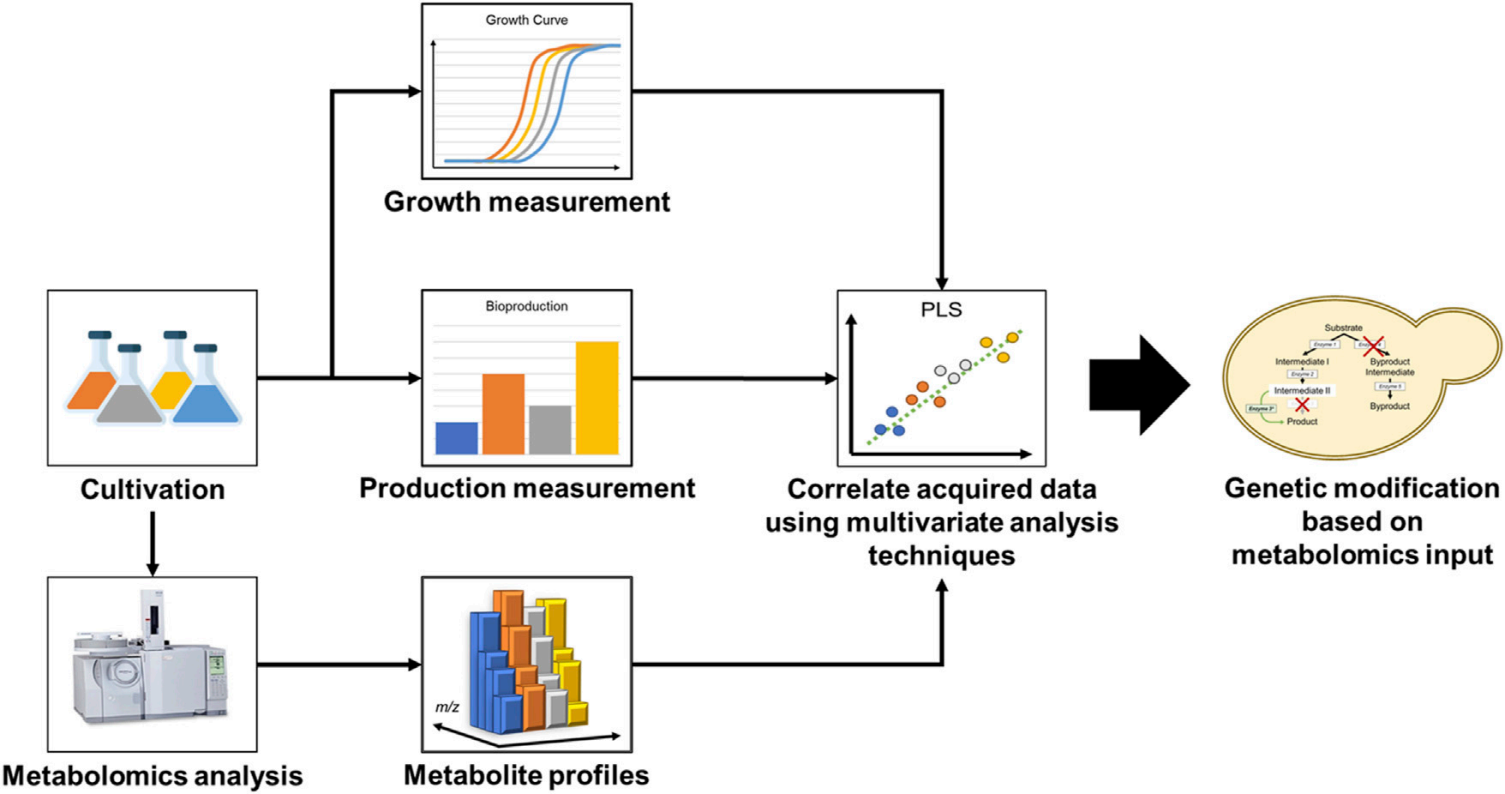
Metabolomics-driven strain improvement workflow.

Metabolomics is a technology that allows for a system-wide quantitative characterization of metabolites. Coupled with statistical multivariate pattern recognition methods, such as principal component analysis (PCA) or partial least squares-discriminant analysis (PLS-DA), metabolomics can be used to acquire deeper insight on cellular metabolome state and investigate how genetic designs affect production phenotype (Putri et al., 2013). For this reason, mass spectrometry-based metabolomics has been established as a powerful and versatile tool in facilitating strain improvement (Nitta et al., 2017). A typical flow of a metabolomics work which leads to inputs for strain improvement is illustrated in Figure 1. Although there are many scientific reports on the use of metabolomics for the elucidation of mechanism and physiological changes occurring in the cell, there are surprisingly very few reports on its use for the identification of rate-limiting steps in a synthetic biological system that can lead to the actual improvement of the host microorganism. In this mini review, we discuss different strategies for improving strain performance using metabolomics data and compare the application of metabolomics-driven strain improvement techniques in different host microorganisms. Finally, we highlight several success stories on the use of metabolomics-driven strain improvement strategies, which led to significant bioproductivity improvements.

## Strategies to improve strain performance using metabolomics data

**FIGURE 2:**
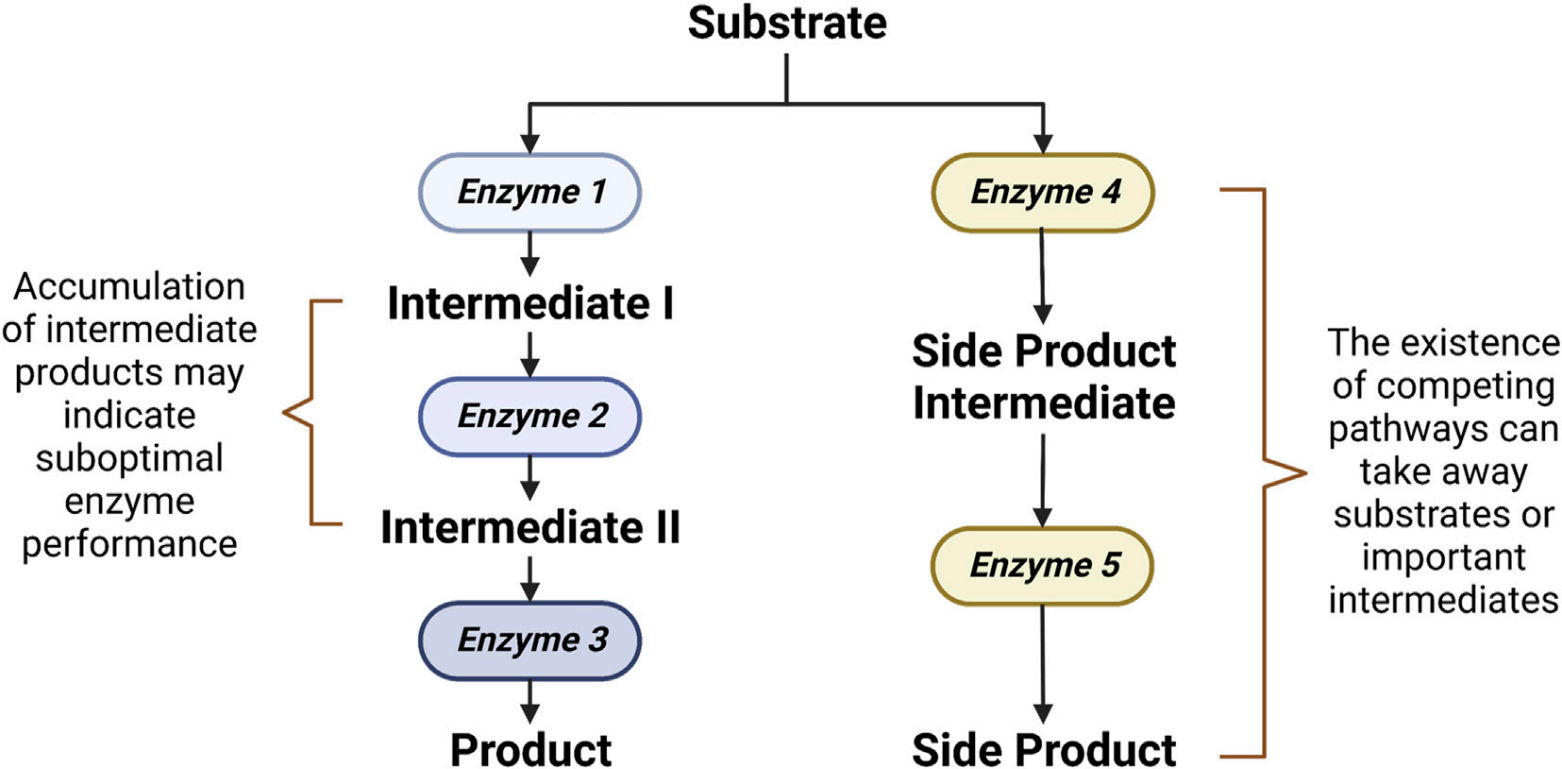
Rate-limiting steps in a synthetic biological system.

Ultimately, the aim of metabolomics-driven strain improvement is the increased bioproduction capability. To this end, the role of metabolomics is to investigate whether the designed system results in a desired production phenotype. Metabolomics allows for the simultaneous monitoring of not only the end product but also the important intermediates along the way (Vignoli et al., 2019). This allows for the accurate pinpointing of the rate-limiting steps or bottlenecks in the bioproduction line, which gives clues on how to further optimize bioproductivity (Hollywood et al., 2018). A bottleneck in a pathway can exist due to either a suboptimal enzyme performance or the existence of competing pathways (Figure 2). A suboptimal enzyme performance is often indicated by an accumulation of intermediates. Gene overexpression, facilitated by either the construction of a ribosomal binding site (RBS) library or a promoter strength adjustment, can be employed to improve a suboptimal enzyme performance (Siegl et al., 2013). On the other hand, the existence of competing pathways which can take away important intermediates can be detected by a metabolic flux analysis (Ghosh et al., 2016). In such cases, knockout of genes in the competing pathways can be applied to reroute the flux (Nitta et al., 2019). Aside from the alleviation of bottleneck reactions, bioproduction can also be improved through the optimizations of cell growth. In some cases, a high concentration of the desired end product or a side product may be toxic and hampers cell growth, which in turn prevents optimal bioproduction. In such circumstances, bioproductivity can be improved by identifying and subsequently optimizing genes that confer stress tolerance (Ohta et al., 2016).

## Metabolome profiling methods

Different experimental designs can be used to facilitate metabolomics-driven strain improvement strategies. In general, however, commonly employed experimental approaches in metabolomics can be categorized into: Targeted metabolomics and non-targeted metabolomics.

Targeted metabolomics focuses on the detection and quantification of a specific, predefined set of metabolites. As a hypothesis-driven approach, targeted metabolomics is ideally used in cases where clues of possible rate-limiting steps are present. For example, Noguchi et al. (2016) employed a targeted analysis to specifically profile acyl-CoAs in *Synechococcus elongatus* PCC 7942 based on the hypothesis that the CoA-dependent pathway may contain a rate-limiting step in 1-butanol production. Common targets for targeted analysis in metabolomics-driven strain improvement studies include metabolites involved in the central metabolic pathway (Kawaguchi et al., 2018; Ohtake et al., 2022) and metabolites surrounding heterologous pathways introduced for bioproduction purposes (Noguchi et al., 2016; Nitta et al., 2019). In targeted metabolomics, chemical standards of target metabolites need to be characterized as references to facilitate accurate metabolite annotation. For this reason, annotation in targeted metabolomics is generally more reliable and reproducible compared to its non-targeted counterpart, despite the coverage being more limited (Cao et al., 2020). Absolute quantification is also possible in targeted metabolomics, which might provide essential insights for understanding a biological system (Nitta et al., 2021).

Non-targeted metabolomics, on the other hand, does not require prior information about specific targets. Non-targeted metabolomics aims to capture as many signals as possible and identify them by cross-referencing obtained spectra with available metabolite spectral databases. In contrast to targeted metabolomics, non-targeted metabolomics is an excellent approach for generating clues of possible rate-limiting steps in a biological production system. In a study, Teoh et al. (2015) employed a non-targeted approach to identify gene targets that might increase 1-butanol tolerance in *Saccharomyces cerevisiae* without prior knowledge of the gene functions. While non-targeted metabolomics provides a wide metabolite coverage and is a powerful tool to examine a biological system, the difficulty of metabolite identification remains to be a crucial limitation (Wasito et al., 2022).

## Metabolomics-driven strain improvements in different organisms

The generation of optimization inputs based on metabolomics data largely depends on the availability of genetic and pathway information of the production host. For some hosts, a complete metabolic map might be available, which may allow for genome-scale metabolic modeling (GSM) (Kavšček et al., 2015). For other chassis, where genetic information is limited, interpretation of the captured metabolomics data may be more challenging. Here, we highlight a number of success stories of applying metabolomics-driven strain improvement strategies in different host organisms, ranging from the model organism *Escherichia coli* to specialized chassis, such as *Aureobasidium pullulans.*

### Canonical hosts: *Escherichia coli* and *Saccharomyces cerevisiae*

Owing to the wealth of genetic information available, characterizations, as well as the molecular toolkit available, *Escherichia coli* and *Saccharomyces cerevisiae* are the two most widely used hosts to produce heterologous metabolites. For this reason, metabolomics-driven metabolic engineering to improve the bioproduction in these two hosts is abundant. Here, we highlight some notable examples of using metabolomics to guide metabolic engineering in *Escherichia coli* and *Saccharomyces cerevisiae.*

A series of studies performed by our group capitalized on metabolomics data to optimize 1-butanol production in *Escherichia coli.* We revealed that the CoA imbalance was due to the deletion of *pta* causing an unwanted accumulation of pyruvate, butanoate, and other CoA-derived compounds. Using metabolomics, the alcohol dehydrogenase AdhE2 catalyzed reduction of butanoyl-CoA to butanal was determined as the rate-limiting step. The refinement of this activity and the subsequent release of free CoA through cysteine supplementation restored the balance of CoA, resulting in a titer of 18.3 g/L. By enhancing the activity of AdhE2, the carbon flux was directed towards the production of 1-butanol and the unwanted accumulation of pyruvate and butanoate was reduced (Ohtake et al., 2017). Meanwhile, optimizing YqhD alcohol dehydrogenase activity using a ribosome binding site (RBS) library improved 1-propanol titer (g/L) and yield (w/g glucose) by 38 and by 29% in 72 h compared to the base strain, respectively for the production of 1-propanol (Ohtake et al., 2022).

A similar strategy was applied to improve the production of 1-butanol in *Saccharomyces cerevisiae.* Growth inhibition due to high alcohol conditions was determined as the bottleneck in 1-butanol production. Accordingly, we capitalized on metabolomics data to improve the 1-butanol tolerance of the producing *Saccharomyces cerevisiae* strain. Non-targeted metabolome analysis using GC/MS coupled with Orthogonal Projections to Latent Structures (OPLS) modeling revealed that threonine and citric acid were among the most important metabolites in conferring 1-butanol tolerance. We proved that individual deletions of genes associated with threonine and citric acid (*met2, cha1, cit2*) lead to higher 1-butanol tolerance (Teoh et al., 2015). Further data mining using Random Sample Consensus combined with Partial Least Squares regression (RANSAC-PLS) on the same dataset suggested that trehalose, valine, and pyroglutamic acid also contribute to the 1-butanol tolerance of *Saccharomyces cerevisiae*. From this input, individual deletions of *xp1*, *bat2*, and *nth1* were performed. This led to higher growth under 1-butanol stress (Teoh et al., 2016).

### Photosynthetic cyanobacterial hosts

Aside from the traditional chassis, cyanobacteria have also been a popular choice for producing biotechnologically significant homologous and heterologous metabolites. The photosynthetic nature of cyanobacteria, its ease of cultivation, as well as its ability to store compounds within its intracellular compartmentalization give cyanobacteria some competitive edge over traditional hosts. On the other hand, metabolomics-driven metabolic engineering often requires ample genetic information and characterizations, which are largely lacking in most cyanobacterial strains. Nonetheless, here, we highlight some success stories of performing metabolomics-driven strain improvements in cyanobacterial hosts.

One example is the widely-targeted metabolomics analysis to improve cyanobacterial 1-butanol yield previously performed by our group. By focusing on the CoA-dependent pathway in *Synechococcus elongatus* PCC 7942, we identified that the reductive reaction of butanoyl-CoA to butanol is a possible rate-limiting step in butanol production (Noguchi et al., 2016). Improved CoA-acylating heterologous propionaldehyde dehydrogenase, which is responsible for this bottleneck step, increased the target compound and free CoA regeneration, leading in turn to increased acetyl-CoA synthesis. The newly discovered rate-limiting step was enhanced by overexpressing heterologous acetyl-CoA carboxylase, resulting in increased 1-butanol levels. We further compared strains that differ in enzymes that convert butanoyl-CoA to butanol, revealing another distinct rate-limiting step in 1-butanol biosynthesis. The result indicated that the reductive reaction of butanoyl-CoA to butanal needs to be further modified to improve both the titer and the productivity of the engineered cyanobacterial strain (Fathima et al., 2018). However, strain sensitivity to 1-butanol itself hindered the production of 1-butanol. Using a high producer of 1-butanol *S. elongatus* DC11 strain, demonstrated a significant accumulation of sugars and nucleosides under salt and alcohol stress compared to the original construction of the strain background. The results obtained from this study may be useful for future strain enhancement strategies in *S. elongatus*, focusing specifically on the metabolic response of this strain to 1-butanol stress (Fathima et al., 2020).

Cyanobacteria have also been explored as promising producers of succinate and D-lactate to develop environmentally friendly, biodegradable plastics. Succinate could be synthesized in PCC 6803 *via* the TCA cycle under dark anoxic conditions; however, it was unclear whether this was achieved through an oxidative or reductive route. Dynamic metabolic profiling of PCC 6803 revealed that succinate is synthesized *via* glycolysis, the anaplerotic pathway and the reductive pathway of the TCA cycle. PCC6803 cultured under dark anoxic conditions, allowing identification of the carbon flow and rate-limiting steps in glycogen catabolism. Glycogen biosynthesized from CO2 assimilated during periods of light exposure is catabolized to succinate *via* glycolysis, the anaplerotic pathway, and the reductive TCA cycle under dark anoxic conditions. Expression of the phosphoenolpyruvate (PEP) carboxylase (*ppc*) gene has been identified as a rate-limiting step in succinate biosynthesis, and this rate limitation has been alleviated by overexpression of *ppc*, resulting in increased secretion of succinate (Hasunuma et al., 2016).

Metabolomics analysis on the recombinant isoprene producing strain *Synechocystis sp*. PCC 6803 revealed that the limitation in isoprene production was due to an insufficient DMAPP level (Pade et al., 2016). Therefore, the mevalonic acid (MVA) pathway, another pathway for the synthesis of DMAPP, was introduced into the isoprene-producing cells to bypass the MEP pathway, resulting in increased isoprene levels. The production of isoprene was also enhanced by the overexpression of the genes encoding Ipi, Dxs and IspD, whose catalytic reactions were identified as bottlenecks within the MEP pathway (Englund et al., 2018).

### Other non-canonical hosts

While less common, there are some studies employing metabolomics-driven metabolic engineering in hosts other than *Escherichia coli*, *Saccharomyces cerevisiae*, or cyanobacteria. As with the case of cyanobacterial hosts, extracting insightful inputs from the obtained metabolomics data might be more challenging in these non-canonical hosts due to the lack of genetic information and characterizations. These hosts are likely selected despite the tradeoffs due to their excellent natural capabilities to produce industrially relevant compounds.

*Corynebacterium glutamicum* is a bacterium with high industrial importance. This bacterium is commonly used in large-scale production of amino acids, most notably L-glutamate. In 2018, Kawaguchi et al. (2018) used metabolomics data input to engineer *Corynebacterium glutamicum* ATCC 31831 strain capable of simultaneous utilization of D-glucose and L-arabinose. LC-MS/MS analysis was used to identify phosphofructokinase and pyruvate kinase as major bottlenecks in the metabolisms of D-glucose and L-arabinose, respectively. Accordingly, they engineered a strain overexpressing pyruvate kinase combined with the deletion of the L-arabinose uptake and catabolism repressor gene, *araR*. This newly improved strain was able to utilize 15 g/L of D-glucose and L-arabinose simultaneously. They also identified citrate synthase to be the new bottleneck in this improved strain during the simultaneous utilization of D-glucose and L-arabinose.

*Clostridium autoethanogenum* is an ethanol producing bacterium capable of utilizing CO and/or CO_2_ + H_2_ gases as its sole carbon and energy sources. In 2019, Lemgruber et al. (2019) engineered and optimized a recombinant *Clostridium autoethanogenum* strain to produce poly-3-hydroxybutyrate (PHB). HPLC-based metabolomics analysis, combined with transcriptomics and genome-scale metabolic modeling, was used to evaluate and further optimize the production of PHB to up to 12 times the original yield.

*Aureobasidium pullulans* is an industrially important fungus commonly used in the production of various enzymes and compounds, including polymalic acid (PMA) and its monomer L-malic acid (MA). In 2018, Feng et al. (2018) used GC/MS-based analysis to perform widely-targeted metabolomics profiling of PMA and MA-producing *Aureobasidium pullulans*. They utilized the multivariate analysis methods of principal component analysis (PCA) and orthogonal-partial least squares-discriminant analysis (OPLS-DA) to recognize insightful patterns and extract information from the obtained metabolomics data. Notably, they also incorporated genome-scale metabolic modelling based on the obtained metabolomics data. Pyruvate metabolism, in particular pyruvate carboxylase (encoded by *pyc*), was identified to be the key metabolite and enzyme affecting PMA synthesis. Based on this input, they engineered a new strain FJ-PYC which over-expresses the *pyc* gene. Owing to this metabolomics-driven metabolic engineering, they were able to increase the PMA yield by over 15%.

## Recent advances and future outlook

While not always accompanied by an immediate demonstration of an increased bioproductivity, recent studies often explore the multidisciplinary integration of metabolomics and other technologies to better understand biological systems and generate suggestions for strain improvement. In a recent study, Webb et al. (2022) developed an approach involving the multi-omics integration of metabolomics, transcriptomics, proteomics, and lipidomics to identify potential targets for strain improvement and successfully demonstrated a 3-fold increase in styrene bioproduction. In another study, Zhang et al. (2022) utilized a combination of metabolomics, genomics, and protein structure simulation to identify F6PPK in the “bifidus” pathway as a vital enzyme to confer osmotic stress tolerance in engineered *Bifidobacterium bifidum*. Metabolomics-driven strain improvement strategies are also often incorporated into a larger, more systematic framework in the Design-Build-Test-Learn (DBTL) pipeline of synthetic biology. The DBTL cycle is a systematic framework designed to accelerate strain improvement processes. Metabolomics-driven strain improvement principles play an integral role in the ‘Test’ and ‘Learn’ stages of this framework.

In the future, the effectiveness of metabolomics-driven strain improvement strategies may be elevated further through the advancements in different facets of metabolomics technology. For instance, more accurate reflection of the actual metabolic state of strains can be achieved through the recent advancements in single-cell metabolomics (Hu et al., 2021) and real-time analysis (Judge et al., 2019). Spatial information of metabolite distribution can also be obtained through the combination of mass spectrometry and imaging techniques (Schleyer et al., 2019; Taylor et al., 2021). Much work is also being done to develop analytical methods to increase the number of metabolites annotated, particularly unstable metabolites with very fast turnover rates (Mousavi et al., 2019). Further technological developments covering these aspects, as well as the expansion of analysis coverage, improvements in data processing algorithms, and the incorporation of automation technologies, would make metabolomics a more powerful technology for strain improvements.
